# Supplementary material for: Human perivascular stem cell-derived extracellular vesicles mediate bone repair
Source: eLife. 2019 Sep 4;8:e48191. doi: 10.7554/eLife.48191 (PMC6764819; doi:10.7554/eLife.48191)
Supplement: Supplementary file 1. [file elife-48191-supp1.docx]

| **Key Resources Table** | | | | |
| --- | --- | --- | --- | --- |
| **Reagent type (species) or resource** | **Designation** | **Source or reference** | **Identifiers** | **Additional information** |
| Mouse | C57BL/6J | Jackson Laboratory | Strain #000664, RRID:IMSR_JAX:000664 | Male, 10 week old |
| Mouse | Pdgfrα-CreER;eGFP transgenic reporter mice | This paper | PMID: 21092857; The Jackson Laboratory, Stock No. 018280 | Male, 10 week old |
| Transfected  construct (human) | PTGFRN shRNA | Genomics Resources in the Hit Center |  | Vector: pLKO.1 |
| Transfected construct (human) | IGSF shRNA | Genomics Resources in the Hit Center |  | Vector: pLKO.1 |
| Recombinant DNA reagent | pLKO.1 (plasmid) | Addgene | RRID:Addgene_10878 |  |
| Antibody | anti-Human CD31-APC-Cy7 (Mouse monoclonal) | Bio Legend | Cat# 303119, RRID:AB_10643590 | FACS (1:100) |
| Antibody | anti-Human CD34-APC (Mouse monoclonal) | BD Pharmingen | Cat# 555824, RRID:AB_398614 | FACS (1:100) |
| Antibody | anti-Human CD45-APC-Cy7 (Mouse monoclonal) | BD Pharmingen | Cat# 557833, RRID:AB_396891 | FACS (1:30) |
| Antibody | anti-Human CD146-FITC (Mouse monoclonal) | Bio Rad | Cat# MCA2141F, RRID:AB_324069 | FACS (1:100) |
| Antibody | anti-Human CD44-AF700 (Mouse monoclonal) | BD Pharmingen | Cat# 561289, RRID:AB_10645788 | FACS (1:20) |
| Antibody | anti-Human CD73-PE (Mouse monoclonal) | BD Pharmingen | Cat# 561014, RRID:AB_2033967 | FACS (1:5) |
| Antibody | anti-Human CD90-FITC (Mouse monoclonal) | BD Pharmingen | Cat# 555595, RRID:AB_395969 | FACS (1:20) |
| Antibody | anti-Human CD105-PE-CF594 (Mouse monoclonal) | BD Pharmingen | Cat# 562380, RRID:AB_11154054 | FACS (1:20) |
| Antibody | anti-human CD9 (Mouse monoclonal) | Santa Cruz Biotechnology | Cat# sc-13118, RRID:AB_627213 | WB (1:200) |
| Antibody | anti-human CD63 (Rabbit monoclonal) | Abcam | Cat# ab134045, RRID:AB_2800495 | WB (1:1000) |
| Antibody | anti-human CD81 (Mouse monoclonal) | Santa Cruz Biotechnology | Cat# sc-166029, RRID:AB_2275892 | WB (1:200) |
| Antibody | anti-human Calnexin (Rabbit polyclonal) | Abcam | Cat# ab75801, RRID:AB_1310022 | WB (1:1000) |
| Antibody | Anti-rabbit IgG, HRP-linked | Cell Signaling Technology | Cat# 7074, RRID:AB_2099233 | WB (1:5000) |
| Antibody | Anti-mouse IgG, HRP-linked | Cell Signaling Technology | Cat# 7076, RRID:AB_330924 | WB (1:5000) |
| Antibody | anti-human CD9 (Mouse monoclonal) | Abcam | Cat# ab2215, RRID:AB_302894 | Neutralizing (1 mg/mL) |
| Antibody | anti-human CD81 (Rabbit polyclonal) | Novus Biologicals | Cat# NBP2-20564, RRID:AB_2811127 | Neutralizing (0.88 mg/mL) |
| Antibody | Mouse IgG1, Kappa Monoclonal [B11/6]-Isotype Control | Abcam | Cat# ab91353, RRID:AB_2811128 | Neutralizing (1 mg/mL) |
| Antibody | Rabbit IgG Isotype Control (Rabbit polyclonal) | Novus Biologicals | Cat# NBP2-24891, RRID:AB_2811130 | Neutralizing (0.88 mg/mL) |
| Antibody | Anti-mouse Ki67 (Rabbit monoclonal) | Abcam | Cat# ab16667, RRID:AB_302459 | IF (1:200) |
| Antibody | anti-mouse Osteocalcin (Rabbit polyclonal) | Abcam | Cat# ab93876, RRID:AB_10675660 | IF (1:100) |
| Antibody | anti-rabbit IgG H&L-AF647 (Goat polyclonal) | Abcam | Cat# ab150083, RRID:AB_2714032 | IF (1:200) |
| Antibody | anti-rabbit IgG H+L-DyLight 594 (Goat) | Vector Laboratories | Cat# DI-1594, RRID:AB_2336413 | IF (1:200) |
| Antibody | anti-mouse IgG H&L-AF647 (Goat polyclonal) | Abcam | Cat# ab150119, RRID:AB_2811129 | IF (1:200) |
| Sequence-based reagent | *GAPDH*_F | This paper | PCR primers | CTGGGCTACACTGAGCACC |
| Sequenced-based reagent | *GAPDH*_R | This paper | PCR primers | AAGTGGTCGTTGAGGGCAATG |
| Sequence-based reagent | *IGSF8*_F | This paper | PCR primers | TACCCCTACATGCATGCCCT |
| Sequenced-based reagent | *IGSF8*_R | This paper | PCR primers | TGGGGAGTAAGGGATCACCG |
| Sequence-based reagent | *RUNX2*_F | This paper | PCR primers | TGGTTACTGTCATGGCGGGTA |
| Sequenced-based reagent | *RUNX2*_R | This paper | PCR primers | TCTCAGATCGTTGAACCTTGCTA |
| Sequenced-based reagent | *SP7*_F | This paper | PCR primers | TGGTTACTGTCATGGCGGGTA |
| Sequenced-based reagent | *SP7*_R | This paper | PCR primers | TCTCAGATCGTTGAACCTTGCTA |
| Sequenced-based reagent | *PTGFRN*_F | This paper | PCR primers | CCTGCAACGTCAGTGACTATG |
| Sequenced-based reagent | *PTGFRN*_R | This paper | PCR primers | AGTCCGCCTTAACAGGATCTC |
| Software | FlowJo | FlowJo | RRID:SCR_008520 |  |
| Software | ImageJ | NIH | RRID:SCR_003070 |  |
| Software | Prism | GraphPad | RRID:SCR_002798 |  |
| Software | CLC Genomics Server | QIAGEN | RRID:SCR_017396 |  |
| Software | CLC Genomics Workbench | QIAGEN | RRID:SCR_011853 |  |
| Software | Partek Genomics Suite | Partek | RRID:SCR_011860 |  |
| Software | Spotfire | Spotfire | RRID:SCR_008858 |  |
| Software | Ingenuity Pathway Analysis | QIAGEN | RRID:SCR_008653 |  |
| Software | Photoshop | Adobe | RRID:SCR_014199 |  |
| Software, algorithm | SPSS | SPSS | RRID:SCR_002865 |  |
| Other | DAPI stain | Vector Laboratories | Cat# H-1500, RRID:AB_2336788 |  |
