# Supplementary material for: Human perivascular stem cell-derived extracellular vesicles mediate bone repair
Source: eLife. 2019 Sep 4;8:e48191. doi: 10.7554/eLife.48191 (PMC6764819; doi:10.7554/eLife.48191)
Supplement: Supplementary file 2. [file elife-48191-supp2.docx]

**Supplementary File 2: Frequency of human PSC in lipoaspirate used.**

| **Patient No.** | **Sample source** | **Human PSC (% of total CD31-/CD45- cells)** |
| --- | --- | --- |
| 1 | Human lipoaspirate | 77.7 |
| 2 | Human lipoaspirate | 63.02 |
| 3 | Human lipoaspirate | 11.84 |
| 4 | Human lipoaspirate | 34.88 |
