# Supplementary material for: Human perivascular stem cell-derived extracellular vesicles mediate bone repair
Source: eLife. 2019 Sep 4;8:e48191. doi: 10.7554/eLife.48191 (PMC6764819; doi:10.7554/eLife.48191)
Supplement: Supplementary file 3. — Yield produced by each PSC-EV isolation are summarized. Protein amounts of harvested PSC-EV were determined by the BCA method. For patient samples 1 and 2, the same cell population was used to isolate EVs at two different passages as indicated. [file elife-48191-supp3.docx]

**Supplementary File 3: Yields of PSC-EV.** Yield produced by each PSC-EV isolation are summarized. Protein amounts of harvested PSC-EV were determined by the BCA method. For patient samples 1 and 2, the same cell population was used to isolate EVs at two different passages as indicated.

| **Human PSC (patient No.)** | **Passage** | **Yield (pg/cell/d)** |
| --- | --- | --- |
| 1 | P4; P6 | 1.144; 1.251 |
| 2 | P4; P7 | 0.896; 1.143 |
| 3 | P9 | 1.328 |
| 4 | P9 | 0.898 |
