# Supplementary material for: Human perivascular stem cell-derived extracellular vesicles mediate bone repair
Source: eLife. 2019 Sep 4;8:e48191. doi: 10.7554/eLife.48191 (PMC6764819; doi:10.7554/eLife.48191)
Supplement: Supplementary file 7. [file elife-48191-supp7.docx]

**Supplementary File 7: Most downregulated pathways among PSC-EV treated BMSC by Ingenuity Pathway Analysis.**

| **Pathway** | **Z score** | **Negative log P value** |
| --- | --- | --- |
| RhoA Signaling | -1.897 | 0.765 |
| Oxidative Phosphorylation | -1.89 | 0.432 |
| Signaling by Rho Family GTPases | -1.5 | 0.864 |
| Th2 Pathway | -1.414 | 0.516 |
| Opioid Signaling Pathway | -1.069 | 0.284 |
| Death Receptor Signaling | -1 | 1.05 |
| Stearate Biosynthesis I (Animals) | -1 | 0.614 |
| 14-3-3-mediated Signaling | -0.816 | 1.38 |
| IL-9 Signaling | -0.816 | 1.34 |
| ErbB2-ErbB3 Signaling | -0.816 | 0.684 |
| GP6 Signaling Pathway | -0.632 | 0.636 |
| Actin Cytoskeleton Signaling | -0.577 | 0.376 |
| ILK Signaling | -0.577 | 0.346 |
| AMPK Signaling | -0.535 | 1.63 |
| Angiopoietin Signaling | -0.447 | 1.09 |
| Retinoic acid Mediated Apoptosis Signaling | -0.447 | 0.58 |
| TREM1 Signaling | -0.447 | 0.408 |
| LXR/RXR Activation | -0.447 | 0.266 |
| IL-3 Signaling | -0.378 | 0.673 |
| G Beta Gamma Signaling | -0.378 | 0.305 |
| PTEN Signaling | -0.333 | 0.608 |
| PI3K/AKT Signaling | -0.302 | 1.29 |
| Insulin Receptor Signaling | -0.302 | 0.991 |
| IL-8 Signaling | -0.258 | 0.963 |
