# Supplementary material for: Human perivascular stem cell-derived extracellular vesicles mediate bone repair
Source: eLife. 2019 Sep 4;8:e48191. doi: 10.7554/eLife.48191 (PMC6764819; doi:10.7554/eLife.48191)
Supplement: Supplementary file 8. [file elife-48191-supp8.docx]

**Supplementary File 8: CD markers enriched in PSC-EVs.**

| **Gene symbol** | **Mean FPKM (PSC-EVs)** |
| --- | --- |
| *CD63* | 5.35428 |
| *CD44* | 4.7948 |
| *CD151* | 4.6063 |
| *CD81* | 4.26103 |
| *CD59* | 4.02719 |
| *CD248* | 3.29756 |
| *CD276* | 3.28692 |
| *CD68* | 3.25812 |
| *CD99_1* | 2.42618 |
| *CD99_2* | 2.39123 |
| *CD320* | 2.29034 |
| *CD164* | 2.15435 |
| *CD99L2* | 1.14869 |
| *CD109* | 0.912184 |
| *CD2AP* | 0.513398 |
| *CD82* | 0.341479 |
| *CD46* | 0.325353 |

FPKM: fragments per kilobasepair per million mapped
