# Supplementary material for: Human perivascular stem cell-derived extracellular vesicles mediate bone repair
Source: eLife. 2019 Sep 4;8:e48191. doi: 10.7554/eLife.48191 (PMC6764819; doi:10.7554/eLife.48191)
Supplement: Supplementary file 9. [file elife-48191-supp9.docx]

**Supplementary File 9: Animal allocation and treatment groups.**

| **Treatment group** | **Dose PSC-EV** | **Injection schedule** | **C57BL/6 Animal #** | **Pdgfrα-CreER; eGFP Animal #** |
| --- | --- | --- | --- | --- |
| PBS Control | - | Percutaneous, twice weekly | 4 | 3 |
| PSC-EV | 1 μg |  | 4 | -- |
|  | 2.5 μg |  | 4 | 3 |
