# Supplementary material for: Human perivascular stem cell-derived extracellular vesicles mediate bone repair
Source: eLife. 2019 Sep 4;8:e48191. doi: 10.7554/eLife.48191 (PMC6764819; doi:10.7554/eLife.48191)
Supplement: Supplementary file 10. [file elife-48191-supp10.docx]

**Supplementary File 10: Antibodies used.**

| **Antibody** | **Company** | **Identifiers** | **Use** |
| --- | --- | --- | --- |
| Mouse anti-Human CD31 | Bio Legend | RRID:AB_10643590 | F |
| Mouse anti-Human CD34 | BD Pharmingen | RRID:AB_398614 | F |
| Mouse anti-Human CD45 | BD Pharmingen | RRID:AB_396891 | F |
| Mouse anti-Human CD146 | Bio Rad | RRID:AB_324069 | F |
| Anti-human CD44 | BD Pharmingen | RRID:AB_10645788 | F |
| Anti-human CD73 | BD Pharmingen | RRID:AB_2033967 | F |
| Anti-human CD90 | BD Pharmingen | RRID:AB_395969 | F |
| Anti-human CD105 | BD Pharmingen | RRID:AB_11154054 | F |
| Mouse anti-human CD9 | Santa Cruz | RRID:AB_627213 | WB |
| Rabbit anti-human CD63 | Abcam | RRID:AB_2800495 | WB |
| Mouse anti-human CD81 | Santa Cruz | RRID:AB_2275892 | WB |
| Rabbit anti-human Calnexin | Abcam | RRID:AB_1310022 | WB |
| Anti-rabbit IgG, HRP-linked Antibody | Cell Signaling Technology | RRID:AB_2099233 | WB |
| Anti-mouse IgG, HRP-linked Antibody | Cell Signaling Technology | RRID:AB_330924 | WB |
| Rabbit anti-mouse Ki67 | Abcam | RRID:AB_302459 | IF |
| Rabbit anti-mouse Osteocalcin | Abcam | RRID:AB_10675660 | IF |
| Mouse anti-human CD9 | Abcam | RRID:AB_302894 | N |
| Mouse IgG1 Isotype Control | Abcam | RRID:AB_2811128 | N |
| Rabbit anti-human CD81 | Novus Biologicals | RRID:AB_2811127 | N |
| Rabbit IgG Isotype Control | Novus Biologicals | RRID:AB_2811130 | N |
| Goat Anti-rabbit IgG(H+L) | Abcam/Vector Laboratories | RRID:AB_2714032  /RRID:AB_2336413 | ICC / IF |
| Goat anti-mouse IgG(H+L) | Abcam | RRID:AB_2811129 | ICC / IF |
| F: Flow cytometry and/or fluorescent activated cell sorting; ICC: Immunocytochemistry; IF: Immunofluorescent staining; N: Neutralizing antibody experiments; WB: Western blot | | | |
