# Supplementary material for: Human perivascular stem cell-derived extracellular vesicles mediate bone repair
Source: eLife. 2019 Sep 4;8:e48191. doi: 10.7554/eLife.48191 (PMC6764819; doi:10.7554/eLife.48191)
Supplement: Supplementary file 11. [file elife-48191-supp11.docx]

**Supplementary File 11: Quantitative PCR primers used.**

| **Gene** | **Forward** | **Reverse** |
| --- | --- | --- |
| *GAPDH* | 5’-CTGGGCTACACTGAGCACC-3’ | 5’-AAGTGGTCGTTGAGGGCAATG-3’ |
| *IGSF8* | 5’-TACCCCTACATGCATGCCCT-3’ | 5’-TGGGGAGTAAGGGATCACCG-3’ |
| *RUNX2* | 5’-TGGTTACTGTCATGGCGGGTA-3’ | 5’-TCTCAGATCGTTGAACCTTGCTA-3’ |
| *SP7* | 5’-CCTCTGCGGGACTCAACAAC-3’ | 5’-AGCCCATTAGTGCTTGTAAAGG-3’ |
| *PTGFRN* | 5’-CCTGCAACGTCAGTGACTATG-3’ | 5’-AGTCCGCCTTAACAGGATCTC-3’ |
