# Supplementary material for: Human perivascular stem cell-derived extracellular vesicles mediate bone repair
Source: eLife. 2019 Sep 4;8:e48191. doi: 10.7554/eLife.48191 (PMC6764819; doi:10.7554/eLife.48191)
Supplement: Supplementary file 12. [file elife-48191-supp12.docx]

**Supplementary File 12: Basic features of human PSCs, ASCs, and BMSCs.**

| **Human cells** | **Isolation techniques** | **Culture techniques** | **Basic morphology** | **FACS phenotype** |
| --- | --- | --- | --- | --- |
| PSCs | Fluorescence-activated cell sorting from human liposuction samples | Growth medium. Medium was changed every 3 d. | Fibroblast-like spindle-shaped morphology | CD34^+^CD146^-^CD31^-^CD45^-^ adventitial cells and CD146^+^CD34^-^CD31^-^CD45^-^ pericytes |
| ASCs | Collagenase Type II (1mg/mL) digestion followed by culture expansion | Growth medium. Medium was changed every 3 d. | Fibroblast-like spindle-shaped morphology | Culture defined cell population (CD44^+^CD73^+^CD90^+^CD105^+^CD31^-^CD45^-^) |
| BMSCs | Isolation of BMSCs from adherent cultures | Growth medium. Medium was changed every 3 d. | Fibroblast-like spindle-shaped morphology | Culture defined cell population (CD44^+^CD73^+^CD90^+^CD105^+^CD31^-^CD45^-^) |

Growth medium: DMEM, 15% FBS, 1% penicillin/streptomycin
